# Repeated hospital admission for intentional poisonings among older adults - a Swedish national register-based study

**DOI:** 10.1186/s12877-024-04717-8

**Published:** 2024-02-15

**Authors:** L Laflamme, E Lindholm, Jette Möller

**Affiliations:** Department of Global Public Health , Karolinska Institutet, Widerströmska huset Tomtebodavägen 18A, 17177 Stockholm, Sweden

**Keywords:** Poisonings, Intentional injury, Hospitalisation, Co-morbidity

## Abstract

**Background:**

Poisoning injuries is an increasing concern among older people, and so is the repetition of intentional poisonings. To date, few studies have documented the pattern and individual risk factors for repeated poisonings. This national study aims to shed light on the burden, pattern, and health-related risk factors of repeated intentional poisoning leading to hospitalization or death among older Swedish adults (50 years and older), with a focus on the year following a first event.

**Methods:**

We conducted a nationwide register-based cohort study of people aged 50–100, hospitalized for intentional poisoning (ICD10: X60-69) during 2006–2016 (*n* = 15,219) and re-hospitalized by poisoning of any intent within a year (*n* = 1710), i.e., up to the end of 2017. We considered in turn, the distribution of the second poisoning in 30-day intervals stratified by intent; poisoning lethality within a month and a year; and the sex-specific association between health conditions and being re-hospitalized for intentional poisoning within one year as compared to being hospitalized only once using logistic regression (odds ratios (OR) with 95% confidence intervals (95% CI)).

**Results:**

Following an intentional poisoning, re-hospitalization within a year was predominantly for a new intentional poisoning (89.7%) and occurred most typically within a month (median 4 days). Death within 30 days occurred in similar proportion for the first and second poisoning (2.3% vs. 2.1% respectively). Among both men and women, comorbidity of psychiatric illness was strongly associated with re-hospitalization for intentional poisoning (adjusted ORs = 1.70; 95% CI = 1.45–2.01 and 1.89 (95% CI = 1.60–2.19) respectively).

**Conclusion:**

Most re-hospitalizations within a year after intentional poisoning are also for intentional poisoning and occur most typically within days. Re-hospitalization is associated with several conditions that are characteristic of poor mental health and there are more similarities than differences between men and women in that respect.

## Introduction

Poisoning, both unintentional and intentional, is a significant public health concern among older people. Studies suggest that the proportion of unintentional poisonings over the total number of poisonings among older people might be on the rise, this applies to some [1–2] but not all [3] contexts. Also, the number– and rate– of intentional poisonings largely surpasses that of unintentional ones at any age among older adults [4–5]. Older people poisoning rises concerns also because of its severity as demonstrated by mortality [6–8] and morbidity [9–10] studies showing that poisoning generally has poorer clinical outcomes among them than it does among their younger adult peers. Poisoning is also known as the most common method of deliberate self-harm and suicide among older adults [3, 6, 10], with drugs being the most common poisoning agent [1, 5–8], 11–13], something that likely reflects a greater access to drugs and elevated risks of improper drug use or adverse drug reaction [14]. Further, while older people are less prone to deliberate-self harm, there is a greater degree of intent and lethality in the act they pose [7, 10, 15], which most often is a self-poisoning. Earlier estimates point to a 4:1 ratio between attempts and completion of in older people compared with between 8:1 and 33:1 for the population as a whole and 200:1 for younger people [7]. An additional dark side of older people’s intentional poisoning is its likely repetition, as is the case for other forms of self-harm [7, 10, 16–17]. Poisoning outnumbers therefore other methods for both incidental and repeated self-harm among older people [18–19]. Age and sex differences among older repeaters have not been much studied but a study from the UK on deliberate self-poisoning and self-injury in older people [20] show an over-representation of men among those aged 60–74 years but not among their older peers (75 + years). It has also been shown that while it is known that repeated poisoning occurs generally within a year, most typically within days [7, 10, 16], the manifold consequences of intentional poisoning have not received much attention, among others subsequent unnatural death (such as accidents, suicides, alcohol intoxications and drug overdoses). Attention has been drawn to this by recent evidence that older people who self-harm may subsequently die from unnatural causes during the following year [15, 19], to a much greater extent than their peers who do not self-harm [15].

As the literature on the repetition, morbidity and mortality of intentional poisoning remains scarce and is relatively dated, population-based studies on recent cohorts of older people are warranted. This national study aims to shed light on the burden, pattern, and health-related risk factors of repeated intentional poisoning leading to hospitalization or death among older Swedish adults (50 years and older), with a focus on the year following a first event.

## Method

### Design

We conducted a register-based cohort study.

#### Study population

The study population was extracted from a national open cohort of individuals born in 1965 or earlier registered as Swedish residents at some point between 2005 and 2016 as per the Swedish Register of the Total Population and aged 50 to 100 years (the population increased from 3 512 142 in 2005 to 3 797 469 individuals in 2016). To identify individuals with first events of hospitalization due to poisoning, we first identified all hospitalizations for poisoning at any point between 2006 and 2016 (*n* = 26,014), and then excluded those hospitalized for poisoning of any intent in the year prior to the study period, i.e., in 2005 (*n* = 171; 0.7%). Following this, we ended up with 15,219 individuals with a first event of intentional poisoning during the study period (2006–2016). These individuals were then followed up for a subsequent hospitalization for poisoning during a one-year time window, i.e., up to 2017 if the first poisoning occurred in 2016.

Swedish national public authorities maintain several high-quality, nationwide registers with the purpose to collect and store a wide range of vital statistics and personal information about the Swedish population. The information can be used for research under specific circumstances and conditions in accordance with Swedish laws and regulations governing data protection, privacy, and research ethics. Information from the registers can be linked using the unique personal identity number assigned to all residents in Sweden [21]. Data was made available to us in such a way that individuals could not be identified. Demographic and health conditions of the study population were followed using information during 2006–2017 (up to 1 year after first intentional poisoning) from the Register of the Total Population [22], and the Longitudinal Integration Database for Health Insurance and Labor Market Studies [23], both maintained by Statistics Sweden and contain information about sociodemographic characteristics (such as age, sex, education, migration status). And further, from the National Patient Register [24], the National Prescribed Drug Register [25], and the Cause of Death Register [26], maintained by the National Board of Health and Welfare and contain health-related information about individuals that have received health care service in Sweden (such as diagnoses, treatments, prescribed medications).

#### Poisoning

All events of poisoning were identified using the National Patient Register, covering all hospitalizations (at least one night) and diagnoses based on the International Classification of Diseases, 10th Revision external cause classification codes (ICD-10) [27]. For the first poisoning event, we focused on hospitalizations due to intentional poisonings ICD-10: X60-X69. Repeated poisoning events (or re-injury for poisoning) was defined as hospitalization due to poisoning occurring within one year after the first intentional one, and where the patient was admitted from either home or from another place of residence. The latter was done to ascertain that the new hospitalization was not due to re-admissions between hospital clinics for the first event. For the repeated poisoning, we considered separate intents, as per ICD-10 classification: intentional (X60-X69), unintentional (X40-X49), and undetermined (Y10-Y19). Date of admission was considered as index date.

#### Health condition

We included the following health conditions as measures of comorbidity (within 30 days before the index date of poisoning) or previous illness (within 365 days before the index date) based on inpatient and outpatient specialized care: psychiatric illness (ICD-10: F00-F99), disease of the circulatory system (ICD-10: I00-I99), musculoskeletal disease (ICD-10: M00-M99), alcohol abuse (ICD-10: E24.4, F10, G31.2, G62.1, G72.1, I42.6, K29.3, K70, K86.0, Y90, Y91, Z50.2, Z71.4, T51), and substance abuse (ICD-10: F11–16, F18, F19, T40, T43.3, Z50.3, Z71.5). We also considered unnatural deaths (ICD-10: X70-X84) that occurred after hospitalization based on cause of death reported on death certificates to the Cause of Death Register.

#### Prescribed medication

The number of prescribed medications was extracted from the National Prescribed Drug Register based on the number of unique dispensed medications (ATC codes at the five-digit level) in the year before the index date and was grouped in five categories: 0, 1, 2–4, 5–9 and 10 medications or more. Polypharmacy was regarded as 5–9 medications and severe polypharmacy 10 or more medications.

#### Demographic characteristics

We considered three demographic characteristics, all attributes determined according to the index year: sex, marital status (married and unmarried), and age group (50–64, 65–79, and 80–100 years).

#### Statistical analyses

We conducted three sets of analyses to characterize the frequency and pattern of repeated severe poisonings, once an intentional one had occurred.

First, we determined how frequently a second poisoning for hospitalization occurred within a year of the first one and, stratifying by intent (intentional, unintentional, undetermined), we compiled the monthly distribution (30-day intervals) of the second hospitalization for poisoning from the index date.

Second, we explored the fatality and potentially related morbidity of intentional poisoning, the former within 30 days and the latter within one year (1-366 days) and considering whether an unnatural death had occurred, as in a recent study [15]. We treated the first and second hospitalization for poisoning separately and stratified the data by sex and age group.

We finally measured the sex-specific association between health conditions and repeated intentional poisoning within a year. These analyses were adjusted for confounding by age and marital status. The association was estimated by odds ratios with 95% confidence intervals using logistic regression.

SAS 9.4 (SAS Institute, 2013, Cary, North Carolina, United States of America) was used for the analyses.

## Results

Of the 15,219 individuals aged 50–100 years who were hospitalized for intentional poisoning during the study period, 1710 (11.2%) were re-hospitalized for a second poisoning event in the following year. Nearly nine in ten of the second poisonings were also intentional (*n* = 1534; 89.7%) while 6.0% were unintentional (*n* = 103) and 4.3% of undetermined intent (*n* = 73). Table 1 presents the characteristics of those re-hospitalized within a year, the focus on the present study, compared to those who were not.

**Table 1:** Characteristics of individuals with two or more intentional poisonings within a year (repeaters) and those with only one (non-repeaters) (2006–2017)

Characteristics	Category	Repeaters ≥ 2 intentional poisonings *n* = 1534		Non-repeaters 1 intentional poisoning *n* = 11,004	
		n	%	n	%
Sex	Men	642	41.9	4715	42.8
	Women	892	58.1	6289	57.2
Age group (yrs)	50–64	1087	70.9	6798	61.8
	65–79	339	22.1	2749	25.0
	80–100	108	7.0	1457	13.2
Marital status	Married	363	24.8	3340	31.9
	Unmarried	1098	75.2	7138	68.1
Comorbidity^**1**^	Psychiatric illness	458	29.9	2075	18.9
	Disease in the circulatory system	99	6.5	711	6.5
	Musculoskeletal disease	67	4.4	443	4.0
Previous illness^2^	Psychiatric illness	961	62.6	4797	43.6
	Disease in the circulatory system	377	24.6	2712	24.6
	Musculoskeletal disease	341	22.2	2117	19.2
	Alcohol abuse	363	23.7	1685	15.3
	Substance abuse	223	14.5	847	7.7
	Intentional injury /not poisoning	906	59.1	5204	47.3
Number of prescribed medications^2^	0	183	11.9	1677	15.2
	1	177	11.5	1515	13.8
	2–4	515	33.6	3852	35.0
	5–9	472	30.8	2866	26.0
	≥ 10	187	12.2	1094	9.9

^1^ 1–30 days before index date

^2^ 1-365 days before index date

**Fig. 1:**
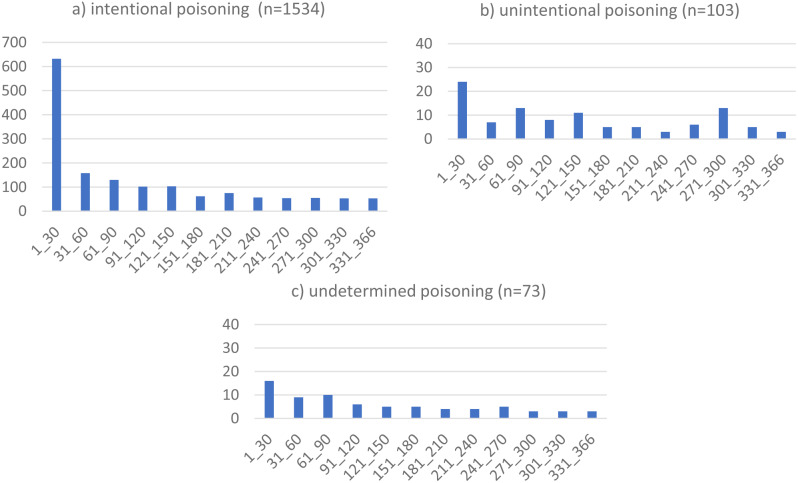
Distribution of re-hospitalization for individuals re-hospitalized for poisoning across 30-day intervals, by intent, following a first hospitalization for intentional poisoning

Figure 1a and 1c show the distribution of the 1710 s poisoning events across 30-day intervals, by intent: intentional (*n* = 1534; 89.9%), unintentional (103; 6.0%), and undetermined (73; 4.1%). Albeit intentional poisonings outnumber by far the two other categories, in all three instances, the second poisoning happens most frequently during the first 30-day interval (41.2%, 23.3% and 21.9% respectively), but the drop in the following months is far more pronounced and consistent in the case of intentional poisoning.

Table 2 shows the 30-days and 1 year all-cause mortality and of other unnatural causes, considering in turn the first and the second hospitalization within a year. In total, the proportion of people who died within 30 days is similar for the first and the second hospitalization for intentional poisoning (2.3% vs. 2.1%) but the share of deaths from unnatural causes is slightly higher for the one-year time window (13.2% vs. 18.8%). In numbers, more people died within one year after the first than the second hospitalization for intentional poisoning (*n* = 159 and 28 respectively) but the proportions are comparable to those observed after 30 days

Similar patterns are observed when stratifying by sex and age. Men exceed women, but the proportions of unnatural deaths are higher among men, most strikingly within 365 days (16.5% compared to 9.6% for death within 30 days and 14.8% compared to 9.0% within 365 days). By contrast, the proportion of those who died after a hospitalization for intentional poisoning increases with age, most remarkably so during the one-year time window (3.6%, 8.3% and 16.7% respectively), but among those who died, dying of natural causes decreased in proportion (and number) with age (19.0%, 9.7% and 5.3%).

**Table 2:** Death all causes and of other unnatural causes 30 days and one year following a first (*n* = 15 219) or a second (*n* = 1534) intentional poisoning, stratified by sex and age

Characteristics		Total number of cases	Death within 30 days				Death within 1 year			
			All causes		Other unnatural cause		All causes		Other unnatural cause	
		n	n	%^1^	n	%^2^	n	%^1^	n	%^2^
**First intentional poisoning**										
Total		15,219	348	2.3	46	13.2	1316	8.6	159	12.1
Sex	Men	6458	182	2.8	30	16.5	702	10.9	104	14.8
	Women	8761	166	1.9	16	9.6	614	7.0	55	9.0
Age group (in yrs)	50–64	9686	136	1.4	24	17.6	492	5.1	91	18.5
	65–79	3717	95	2.6	12	12.6	403	10.8	42	10.4
	80–100	1816	117	6.4	10	8.5	421	23.2	26	6.2
**Second intentional poisoning**										
Total		1534	32	2.1	6	18.8	146	9.5	28	19.2
Sex	Men	642	17	2.6	3	-	74	11.5	18	24.0
	Women	892	15	1.7	3	-	72	8.1	10	13.9
Age group (in yrs)	50–64	1087	20	1.8	4	-	79	7.3	19	24.0
	65–79	339	8	2.4	2	-	48	14.2	5	10.4
	80–100	108	4	3.7	0	-	19	17.6	4	-

^1^% of total number of cases

^2^% of total number of all cause deaths during the period

- % only presented when the total number of deaths is 30 and above

Table 3 presents the results of the analyses on the association between health conditions and prescribed medications among those re-hospitalized for intentional poisoning (within one year) compared to those who were not re-hospitalized. Psychiatric illness is a comorbid condition strongly associated with re-hospitalization for intentional poisoning within a year among both men and women, after adjustment for age and marital status (adjusted ORs = 1.70; 95% CI = 1.45–2.01 and 1.89; 95% CI = 1.60–2.19 respectively) and the only one among women. Among men, after adjustment, even disease of the circulation system is associated (adjusted OR = 1.45; 95% CI = 1.10–1.94).

By contrast, among men, disease of the circulation system is the only previous illness measure that, after adjustment for age and marital status is not associated with a risk of re-hospitalization for intentional poisoning (adjusted OR = 1.17; 95% CI = 0.97–1.41). Otherwise, all considered illnesses during the year prior to index date are strongly associated with re-hospitalization among both men and women, including psychiatric illness but even substance abuse, alcohol abuse, and intentional injury other than poisoning.

Further, after adjustment for age and marital status, compared to not being prescribed medications in the year prior to the index date, the number of prescribed medications showed a significant association with being re-hospitalized for intentional poisoning. For men, a significant association was found for both polypharmacy (5–9 medications) (adjusted OR = 1.44; 95% CI = 1.12–1.87) and severe polypharmacy (10 medications or more) (adjusted OR = 1.49; 95% CI = 1.07–2.07) and for women, for 2–4 medications (adjusted OR = 1.51; 95% CI = 1.16–1.94), 5–9 (adjusted OR = 1.97; 95% CI = 1.52–2.55) and 10 or more (adjusted OR = 2.30; 95% CI = 1.71–3.10).

**Table 3:** Association between health conditions and prescribed medications and being re-hospitalized for intentional poisoning within one year as compared to being hospitalized only once, crude and adjusted odds ratios (OR) with 95% confidence intervals (95% CI), stratified by sex

Health conditions	Men *n* = 642		Women *n* = 892	
	OR (95% CI) Crude	OR (95% CI) Adjusted^1^	OR (95% CI) Crude	OR (95% CI) Adjusted^1^
**Comorbidity** ^**2**^				
Psychiatric illness Disease of the circulatory system Musculoskeletal disease	1.90 (1.62–2.29) 1.22 (0.93–1.61) 1.08 (0.73–1.62)	1.70 (1.45–2.01) 1.45 (1.10–1.94) 1.14 (0.75–1.74)	2.00 (1.73–2.35) 0.96 (0.71–1.30) 1.21 (0.88–1.66)	1.89 (1.60–2.19) 1.18 (0.85–1.63) 1.27 (0.90–1.76)
**Previous illness** ^**3**^				
Psychiatric illness Disease of the circulatory system Musculoskeletal disease Alcohol abuse Substance abuse Intentional injury other than poisoning	2.12 (1.79–2.50) 1.01 (0.85–1.20) 1.26 (1.03–1.53) 1.84 (1.55–2.18) 1.86 (1.49–2.32) 1.79 (1.52–2.12)	1.90 (1.59–2.26) 1.17 (0.97–1.41) 1.28 (1.04–1.57) 1.62 (1.35–1.94) 1.70 (1.35–2.14) 1.85 (1.56–2.20)	2.49 (2.17–2.87) 1.08 (0.92–1.27) 1.26 (1.07–1.48) 2.17 (1.81–2.60) 2.69 (2.19–3.30) 1.78 (1.55–2.04)	2.29 (1.98–2.66) 1.26 (1.06–1.51) 1.27 (1.08–1.50) 1.90 (1.58–2.29) 2.45 (1.98–3.02) 1.85 (1.60–2.13)
**Number of prescribed medications** ^**3**^				
0 1 2–4 5–9 ≥ 10	1.0 (REF) 0.92 (0.68–1.25) 1.03 (0.81–1.31) 1.28 (0.99–1.64) 1.22 (0.87–1.68)	1.0 (REF) 0.97 (0.71–1.33) 1.12 (0.86–1.42) 1.44 (1.12–1.87) 1.49 (1.07–2.07)	1.0 (REF) 1.22 (0.90–1.65) 1.51 (1.17–1.94) 1.91 (1.48–2.46) 2.12 (1.58–2.83)	1.0 (REF) 1.25 (0.91–1.70) 1.51 (1.16–1.94) 1.97 (1.52–2.55) 2.30 (1.71–3.10)

^1^ Adjusted for age and marital status

^2^ 1–30 days before index date

^3^ 1-365 days before index date

## Discussion

The main findings of this study are that most but not all re-hospitalizations for poisoning within the year following an intentional poisoning are also due to an intentional poisoning and that rehospitalization occurs most typically within days. Repeated intentional poisoning is slightly more common among women than men and far more common among the younger group of older people (50–64 years). Within the subsequent 30 days, a small and similar proportion of the first and second intentional poisoning are followed by a death report, some of those deaths were from unnatural causes but in lower proportions than in the case of death within a year. Further, there are more similarities than differences in the health conditions of intentional poisoning repeaters and non-repeaters. Psychiatric illness is strongly associated with re-hospitalization, be it measured as a comorbidity or as apparent during the year preceding the hospitalization. Other such significant apparent health conditions are substance abuse, alcohol abuse, intentional injury other than poisoning, and polypharmacy and severe polypharmacy. For women, even being prescribed less medications [1–2, 5] is also associated with an excess odds of re-hospitalization.

### Occurrence of self-poisoning and subsequent death

To the best of our knowledge, studies on the repetition of self-poisoning specifically among older people are uncommon, which makes comparisons difficult, but there are some studies on the repetition of self-harm in that population [19]. Whereas the short time lag between two consecutive hospitalizations could be expected [7, 10, 16–17, 19], few previous studies have shown that rapid re-hospitalization for undetermined and unintentional poisoning may also occur. The monthly distribution of repeated intentional poisoning observed herein aligns well to that observed in Hong Kong at population level when studying non-fatal self-harm all methods aggregated (2002–2016; [18]), and so do the sex and age differences. Interestingly, in the latter study, previous self-injury combined with self-harm was a greater risk factor of self-harm repetition than self-injury alone.

The age and sex differences we observed among repeaters echo those observed in the Hong Kong study at population level (x) and in the UK for older people [20]. We have investigated whether sex differences differed with age, which was the case in the UK study [20]. Finally, albeit not specifically based self-poisoning but rather on self-harm, some earlier studies have also documented the link between self-harm and subsequent suicide alone [19] and that with suicide or other unnatural death in the following year [15].

### Health condition

Further, poor physical illness and mental health condition among older people are known risk factors for self-poisoning, the former association being plausibly mediated through depressive symptoms [7]. For their part, previous self-harm itself and previous psychiatric treatment are acknowledged independent risk factor for self-arm repetition [19]. Whereas studies on the health-related risk factors for re-hospitalization for self-harm among older people are few [19], there are indications that a positive blood alcohol concentration [17], substance misuse [18] and being already receiving mental services [17–18] at time of self-injury increase the risk of repeating self-harm or committing suicide within a year [17]. This is in line with our findings for both men and women, although the measures and time windows we used for mental health are different. It is also of note that number of medications is strongly associated with re-hospitalization for intentional poisoning within one year. This may reflect the poor health condition of the people concerned alongside their greater access to drugs and a related elevated risk of overdose [7]. It is also known that drugs associated with drug overdose among people are more toxic than those found among their younger peers who self-poison [9]. Whether the drugs used for an incidental and repeated poisoning differ had not been documented.

### Strengths and limitations

In spite of the vulnerability of the older segment of the population, studies on the repetition of intentional self-poisoning are few and so are population-based studies on more recent cohorts of older people. The strength of our study lies in the implementation of a cohort study based on register-information covering the whole population. This minimizes the risk of selection bias and misclassification and provides good insight into the magnitude of the problem. We have identified all diagnosed hospitalized events of poisoning and linked them to demographic and health data, allowing for adjustment for confounding. We included several measurements of health conditions, including common major morbidities, however we did not have access to health conditions mainly diagnosed and treated in primary care settings and there might be less severe comorbidities or acute illnesses (such as infections) that play a role for the risk of poisoning, particular non-intentional ones during the follow-up. Further, events of poisoning were based on hospitalizations, and we did not have information about poisoning events that were less severe and hence not treated at hospital, however equally so for the first and repeated poisoning events. We acknowledge that the intent could have been misclassified by the clinician. It is common in studies of suicides to include undetermined events as intentional [28]. In this study, however, we limited the first event of poisoning to those coded as intentional. This might lead to an underestimation of the magnitude of the problem and limit the generalizability.

Although this study was based on a large cohort of older adults, statistical power was limited in some of the stratified analyses, specifically when assessing fatality within time periods. Additionally, the analyses could not be stratified on the actual substance– or combinations of substances– causing the poisoning. However, this is likely to be related to medications [29] but may differ between repeaters and non-repeaters. As rightfully pointed by others, index episodes of incidental self-harm in studies like the ones we conducted are not generally lifetime first episodes [30]. As a consequence, follow-up data are based on an arbitrary starting point.

### Implications for prevention

Owing to the facts that older people who self-harm is at very high risk of either repeated self-harm or suicide [17] and that poisoning is, among them, the most common form of incidental and repeated self-harm, self-poisoning in this population group deserves particular clinical and public health attention [7]. Medical professionals certainly have a key role to play in the recognition of groups at risk of self-harm and self-poisoning repetition, fatal or not [14].

Preventive measures of self-harm repetition put forward in the literature include having mental health professionals present in hospital emergency departments; improved social and health alert actions [31], careful alternative prescribing, including reduced exposure to drugs associated with significant toxicity in general [9] and benzodiazepines in particular [15, 32], screening and assessment for alcohol use disorders [17], and increased support to enhance resilience among older adults who consult after an episode of self-harm or those with known comorbidities [15]. To that end, it is recommended to adopt strategies that combine structured psychological and tailored pharmacological treatment [17].

## Conclusion

Most re-hospitalizations within a year after intentional poisoning are also for intentional poisoning and they occur most typically within days. They are slightly more common among women and far more common among younger older people (50–64 years). Death from unnatural causes is more common within a year than within 30 days.

Re-hospitalization is associated with several conditions that are characteristic of poor mental health and there are more similarities than differences between men and women in that respect.

## Data Availability

The data that support the findings of this study are available from the Statistics Sweden and the National board of Health and Welfare, but restrictions apply to the availability of these data, which were used under license for the current study, and so are not publicly available. Data are however available from the corresponding author (JM) upon reasonable request and with permission of the Statistics Sweden and the National board of Health and Welfare.
